# Evaluating Synthetic Medical Images Using Artificial Intelligence with the GAN Algorithm

**DOI:** 10.3390/s23073440

**Published:** 2023-03-24

**Authors:** Akmalbek Bobomirzaevich Abdusalomov, Rashid Nasimov, Nigorakhon Nasimova, Bahodir Muminov, Taeg Keun Whangbo

**Affiliations:** 1 Department of Computer Engineering, Gachon University, Sujeong-Gu, Seongnam-Si 461-701, Gyeonggi-Do, Republic of Korea; 2 Department of Artificial Intelligence, Tashkent State University of Economics, Tashkent 100066, Uzbekistan

**Keywords:** echocardiogram, artificial intelligence, echocardiography, generative adversarial networks, convolutional neural network, FID, FMD, IS, synthetic medical image

## Abstract

In recent years, considerable work has been conducted on the development of synthetic medical images, but there are no satisfactory methods for evaluating their medical suitability. Existing methods mainly evaluate the quality of noise in the images, and the similarity of the images to the real images used to generate them. For this purpose, they use feature maps of images extracted in different ways or distribution of images set. Then, the proximity of synthetic images to the real set is evaluated using different distance metrics. However, it is not possible to determine whether only one synthetic image was generated repeatedly, or whether the synthetic set exactly repeats the training set. In addition, most evolution metrics take a lot of time to calculate. Taking these issues into account, we have proposed a method that can quantitatively and qualitatively evaluate synthetic images. This method is a combination of two methods, namely, FMD and CNN-based evaluation methods. The estimation methods were compared with the FID method, and it was found that the FMD method has a great advantage in terms of speed, while the CNN method has the ability to estimate more accurately. To evaluate the reliability of the methods, a dataset of different real images was checked.

## 1. Introduction

In the last few years, the use of artificial intelligence (AI) to analyze images, videos, text, and audio, in order to interpret, detect, classify, and diagnose diseases, has attracted the growing interest of researchers [1,2]. The development of medical AI-based software requires a huge amount of data such as blood test results, X-rays, Computed tomography (CT), Magnetic resonance imaging (MRI) Echocardiography (Echo) images, etc. However, developing and labeling such datasets is a costly and time-consuming process, as those processes are usually carried out by human experts. 

Currently, there are limited numbers of publicly available echo databases, which are too small or big but unlabeled. Therefore, there is a high demand for synthetic image-creation methods. So, in recent years, building synthetic echocardiogram image datasets has received considerable attention [3,4,5]. Generative adversarial networks (GAN) [6], autoencoders (AEs) [7], and U-nets [8] have become the most efficient and popular methods to generate synthetic images, and hundreds of their hybrid algorithms have been proposed so far [9,10].

Obviously, as the number of algorithms increases, the need to evaluate their quality and reliability also grow, respectively. Traditional methods of image quality assessment were used usually to evaluate synthetic medical images. However, natural images (i.e., images of people, cars, animals, and other things around us) are slightly different from medical images. 

Firstly, natural images are normally RGB images, i.e., 2D with 3 channels, while medical images can be presented in different forms, such as 2D Gray scale (1 channel), 2D with 4 channels, 3D, and 4D. Secondly, the relative pixel intensity in the natural image is determined to detect edges and gradients. Quite in reverse, the intensity of each pixel in medical images can convey information relevant to the problem, and even noise can provide information about pathologies in tissues or organs. For example, algorithms for detecting fibrosis have been developed based on speckle noises in echocardiogram images [11]. Whereas in normal images, speckle noises are considered unnecessary and are tried to be reduced as much as possible. Thirdly, in medical images, variation in location plays a crucial role, but the location of an object is not important in normal images.

For this reason, in works [12,13], it is shown that using transfer learning with famous networks (i.e., Inception, Resnet, VGG) to classify medical images does not give good results, as those networks are only trained on natural images. Similarly, the methods used to evaluate natural images cannot be used to evaluate medical images. Because popular image quality evaluation methods such as FID, IS uses the Inception network to evaluate images. However, in recent studies, the FID measurement was used as an evaluation method [14,15,16], even though this leads to incorrect evaluation of image quality. Moreover, calculating the FID value takes a lot of time and memory. It is especially disruptive when it is used as an additional loss in training GAN networks. Therefore, it is an important task to develop an easy-to-compute evaluation method specifically designed for medical images. In recent years, special evaluation methods have been proposed for MRI, CT, and PET, but these methods cannot be used directly to evaluate artificial echocardiogram images. Despite this, any special method has been proposed in this field so far.

Moreover, FID or IS score cannot distinguish very subtle differences in echo images. Here is an example to explain it better. The first sign to diagnose Hypertrophic cardiomyopathy (HCM) based on an echocardiogram is the LV thickness assessed at the level of the septum and free wall. More precisely, if it is 15 mm or thicker, it means that this patient may have HCM. The ratio of septal to free wall thickness is equal to 1.3–1.5 and is also considered a suspicious sign of HCM [11]. 

**Figure 1:**
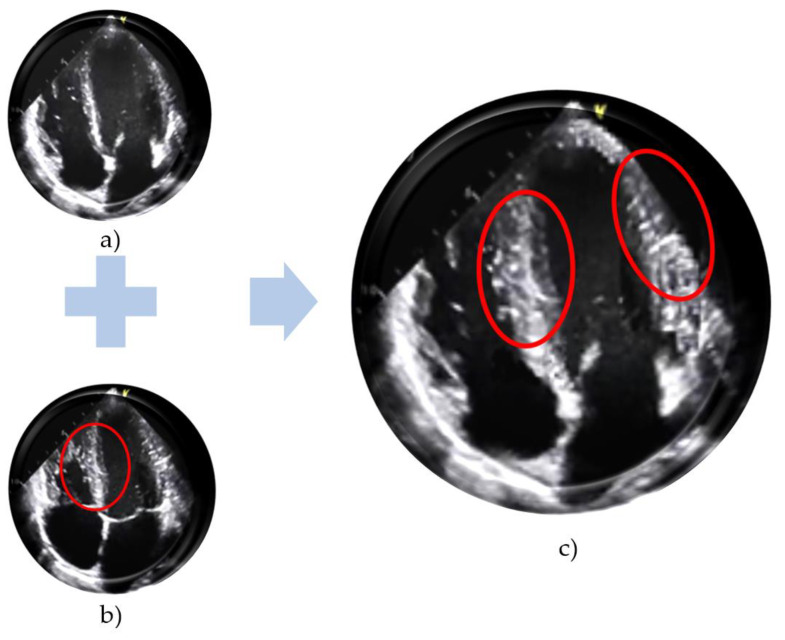
From the image of a healthy heart when its mitral valves open (**a**) and closed (**b**), a synthetic heart image with the open mitral valve and thickened LV (**c**) is made. (Figure (**a**,**b**) are taken from [17], while (**c**) is made manually.)

However, since the LV value usually changes with the contraction and expansion of the heart, this value should be measured during mid-diastole. That is, the ventricle of a healthy heart expands during diastole (it will be largest when the mitral valves are closed (Figure 1a) and narrows during the systole (it will be narrowest when the mitral valves are open (Figure 1b). For this reason, the LV value is measured in the middle of diastole time. Now just imagine if the training dataset consists of healthy patients’ echo images. Naturally, this dataset includes images of diastolic and systolic phases of the cardiac cycle. The image generator takes the details/features from these two real images and generates a new one (an image of the heart with an LV as wide as an end-diastolic LV but with open valves can probably be generated) (Figure 1c.). While this synthetic image is considered a high-quality image by most evaluation criteria, it actually does not describe the target disease, but it describes another disease, i.e., in the abovementioned case, it represents HCM signs. 

Therefore, in order to fill this gap, a new evaluation approach was proposed in this paper. A brief summary of the proposed method is provided below. The assessment is carried out in two stages:

1. Quantitative evaluation: the assessment of the quality (noise, similarity) of the images.
2. Qualitative evaluation: the assessment of the reliability of the images.

It was proposed to use a slightly modified version of the Fréchet inception distance (FID) metric to evaluate the quality of images and it was named the Fréchet MedicalNet distance (FMD) score. The main advantage of the proposed metric is low calculation time compared to the FID score, so it can be used as an additional loss coefficient of the discriminator during training GAN networks and can prevent the gradient from vanishing. This can save a lot of time compared to using a simple FID. This is discussed in detail in paragraph four.

Synthetic data consists of artificially generated data and is a quite powerful tool to overcome the aforementioned problems. Because synthetic data are generated rather than collected or measured, they can be of much higher quality than real data. Moreover, privacy constraints can be applied so that the synthetic data does not reveal any important information, such as patients’ clinical records. The deep neural network was used as a major tool to evaluate the reliability of the synthetic images. In order to train this network, synthetic images of two different classes are used as training datasets, whereas real images are used as validation datasets. Network accuracy and convolution matrix are used as the main evaluation parameters. 

This paper consists of the following sections. The second section presents and analyzes the related works. The third section describes image generation processes. The fourth section contains information about the proposed methods, related architecture, mathematical apparatus, and obtained results. The fifth section discusses the proposed synthetic image evaluation approach. Finally, the sixth and the seventh are future work and conclusion, respectively.

## 2. Related Work

GANs are a powerful deep generative model trained with an adversarial procedure. GANs have undergone several modifications since they were first proposed to solve several different problems in different domains, e.g., physics [18], healthcare [19], or object detection [20]. To analyze the state-of-the-art in what concerns GANs used for synthetic data generation, as well as synthetic data generation methods, we reviewed recently published scientific papers [21,22,23]. Pose-driven attention-guided image generation for person re-Identification proposed in [24] by Amena et al. introduces attentive learning and transferring the subject pose through an attention mechanism based on GAN. In [25], the study aimed to synthesize artificial lung images from corresponding positional and semantic annotations using two generative adversarial networks and databases of real computed tomography scans. researchers implemented an efficient strategy for synthesizing artificial CT lung images from annotation masks and semantic labels and assessing the quality and realism of the generated images. To create a collection of artificial one-, two-, and four-cell embryo images, generative adversarial networks were trained on real human embryo cell images. The algorithm’s ability to manipulate the size, position, and quantity of artificially generated embryo cell images was confirmed by the results. These images can then be used to train and validate additional embryo image processing algorithms when real embryo images are not available or when the number of real embryo images is insufficient for neural network training [26].

The development of different new methods of generating synthetic images has made it urgent to evaluate these methods, more precisely, evaluating the quality of the image they produced. For this reason, metrics were proposed to estimate synthetic image quality. However, medical images such as MRI, CT, and Echo images are usually different from typical images (i.e., from images of people, cars, animals, clothes, etc.). They are noisier, blurry, and difficult to detect edges and features in the images, especially echocardiogram images. Every little change in them can be a sign of some diseases. So, special and accurate evaluation metrics need to assess the quality of synthetic medical images. A number of papers have been published in recent years to meet this need. They proposed different methods and metrics. The advantages and disadvantages of the methods are given in Table 1.

In [27,28,29], a review and deep analysis of the evaluation methods of synthetic images were given, and important analytical conclusions about their disadvantages and advantages were drawn. When generative models began to be used in healthcare, the simplest Image Quality Assessment methods were first used to evaluate the quality of the generated images. The most popular of them were methods such as Structural similarity index measure (SSIM) [30], Mean squared error (MSE) [31], Mean absolute error (MAE) [32], Peak signal-to-noise ratio (PSNR) [33]. However, these algorithms were very simple and insufficient. For example, when calculating MAE and SSIM, matching pairs of images are required for synthetic images to compare.

As it is impossible to find real and synthetic paired images, many authors [34] used MAE to evaluate generated CT, MRI, or PET image quality. These images are 4D images; thus, their GAN network generated slices of the 4D images. Additionally, some authors proposed to calculate the MAE value between images of successive slices [32]. Although this method can be used to evaluate synthetic MRI and CT images, it is impossible to use them for echo images as well because the echo images are 2D images and they do not have slices. In addition, this method cannot evaluate the proximity of the generated image to real ones, which is one of the method’s considerable disadvantages [35].

**Table 1:** Related works pros and cons.

Name of Method/Metric	Showed High Accuracy Only In	Advantage	Disadvantage
MAE/MSE/SSIM [30,31,32]	Synthetic PET/CT/MRI images	Has high accuracy in assessing noise in images	Require reference image for each synthetic image, Cannot be used for assessing echocardiogram synthetic image
NIQE [36]	Image quality assessment	Do not require reference image for each synthetic image	It can only correctly evaluate noisy synthetic images. Cannot evaluate better quality synthetic images with high accuracy.
IS [37]	Natural images assessment	Do not require reference image for each synthetic image.	It can only evaluate the distribution of generated images. Adapted to the evaluation of natural images;
FID [15]	Natural images assessment	It can estimate the distance between the distribution of generated image set and that of real image set.	Long calculation time; Adapted to the evaluation of natural images;
FastFID [16]	Natural images assessment	Fast calculation time	Adapted to the evaluation of natural images;
DQA [38]	MRI images	Higher evaluation accuracy	Adapted to the evaluation of MRI images;
HYPE [39]	Medical and natural images	Has highest accuracy; Used as a gold standard;	Costly and time consuming
**Proposed Method**	Echocardiogram images	Fast and reliable	Combination of two methods

All the abovementioned methods require reference/ground truth. Naturally, it is impractical to find such an image, because generative models generate images that have a multivariate statistical relationship with a set of real images, but not twins of real images. Therefore, non-reference methods, such as the naturalness image quality evaluator (NIQE), began to be used with a combination of other methods [36]. The statistical distribution of the image is evaluated not by that of another base image but by calculating the deviations from the statistical regularities of the image itself. Because of this, it cannot evaluate the similarity between the real set and the synthetic set, it can only evaluate the quality of the image. However, in the work [36], it was noted that the use of the NIQE method in the earlier epochs of GAN, gives good results. As in the initial epochs of the training, the images will be of poor quality and slightly noisy.

Later, many methods based on measuring the similarities/distances between statistical distributions in a set of images or the distances in different feature maps of real and synthetic images were used (for example, deep quality assessment (DQA) metrics [38], learned perceptual image patch similarity (LPIPS) [40], inception score (IS) [37], and FID scores) [15]. IS score cannot assess the diversity of the images set, i.e., the exact or same images generated by saturated, overfitted or mode collapsed network will be overestimated. It only takes into account the distribution of synthetic images and cannot assess distances between the distribution of real and synthetic image sets. Moreover, it is an image resolution-sensitive method. Furthermore, when compared to human expert evaluation, it shows an unsatisfactory evaluation ability for medical images. Further drawbacks can be found in [28]. Therefore, in recent years, this method is considered unsatisfactory for use in the evaluation of medical images and is almost not used.

Fid was suggested by Heusel [14] as an alternative to IS. Unlike IS, it could also estimate the similarity of images to real images using the distance between activation distributions of datasets obtained from a special layer of the InceptionV3 network. The FID was shown good correlation with human visual perception [30]. For this reason, FID values are used mainly in the evaluation of medical images recently [14,15,16]. However, it still has a number of drawbacks; the most important is high bias. Additionally, FID cannot detect the GAN that remembers the training set.

In addition to the above automated methods, evaluation approaches, which involve humans/experts, have also been used, for example, the visual turing test [41], five-point Likert scale [38], and human eye perceptual evaluation (HYPE) [39]. Although these methods are considered the most accurate methods and are the gold standard, they are costly and time-consuming.

## 3. Image Generation Processes

### 3.1. The Working Principle of the GAN

**Figure 2:**
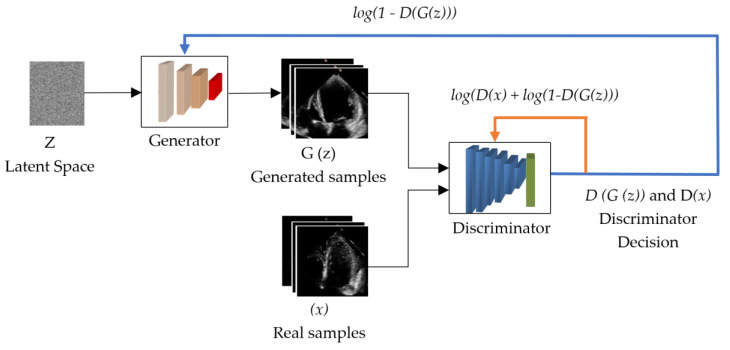
The general architecture of the GAN network.

One of the research fields in medical image processing is generating synthesized images based on generative adversarial networks (GANs). GANs are a framework that uses an adversarial process to estimate generative deep learning models, proposed by Ian J. Goodfellow et al. [42] in 2014. The GAN architecture was named the most interesting idea of the decade. In fact, it was capable of producing sharper, brighter, and more realistic images than AE, U-Net, or other generative networks. One of its advantages was the high level of diversity of the images it produced. The GAN architecture consists of two networks which compete with each other. The architecture of the general GAN is illustrated in Figure 2. This architecture consists of typical generator and discriminator networks. 

A generative model G tries to generate images similar (but not identical) to the real target set from uniformly distributed noise, such as Gaussian noise. While discriminative model D tries to distinguish the generated image from the real one, i.e., determines whether the generated image is synthetic or natural. The total loss function of this network (min-max loss function) is determined using Equations (1) and (2) as follows:

(1) $$\frac{\min max}{GD}V(G,G)$$

(2) $${V\left(D,G\right)=E}_{x\sim P{data}^{\left(x\right)}}\left[logD\left(x\right)\right]+E_{z\sim P_{z}\left(Z\right)}\left[\log\left(1-D\left(G\left(z\right)\right)\right)\right]$$

where D(x) is the discriminator that evaluates the probability that the given data x is real, $E_{x}$ is the expected value for all true datasets, $G\left(z\right)-$ z is the image formed at the output of the generator when noise is given, $D(G\left(z\right))$ is the discriminator that evaluates the probability that the synthetic image is real, and $E_{z}$ is the expected value of the result of all random noise entered into the generator (in fact, the expected value of all generated synthetic examples is $G\left(z\right)$).

From the formula, it is clear that generator losses do not directly affect the network; an increase in generator losses leads to a decrease in total losses because, as the generator loss increases, the generated image quality decreases and becomes noisier. Then, discriminator will easily distinguish such poor-quality images from real ones. Training this network is very challenging. One main issue is the vanishing gradient. As the discriminator’s classification ability increases, the loss value it transmits to the generator becomes very small and the gradient loss function approaches zero: ${1-logD(G}_{z})\approx 0$. As a result, the generator receives no information, and the learning process is terminated. When such a problem occurs, it can usually be overcome using other loss functions instead of adversarial loss, such as Wasserstein loss and their combination [43,44,45,46,47] or adding additional loss to the main loss function as a penalty.

In recent years, many papers have proposed various loss functions [9,47], such as $L_{adversarial}$,$L_{image}$, $L_{perceptual}$, $L_{structure}$ [46], $L_{self-reg}$, $L_{sharp},$ and $L_{shape}$ [9] can be added to the main loss function or can be used in different combinations or instead of it. However, many of them are difficult to calculate, or impossible to reuse and check because the authors do not provide complete information about them, or use private datasets. Therefore, the simplest method is to use the FID score as a loss penalty. In order to find the FID value, the root mean square of the matrices and traces should be calculated, and the calculations are very time-consuming and slow down the process of training the network. Especially during the generation of high-resolution synthetic images from large datasets, adding the FID value as an additional loss further complicates the training process.

### 3.2. GAN Architecture and Parameters

In this work, we use a typical GAN network to evaluate the echo images generated by the GAN network. In the GAN architecture, the generator network consists of five blocks, each of which consists of a successive Convolution Transpose layer, Batch Normalization, and ReLU layers, only the last Convolution Transpose layer is followed by the Tanh layer, instead of Batch normalization, and ReLU layers. The Discriminator network consists of five consecutive Convolution blocks, which include the Convolution layer, Batch normalization, and LeakyReLU layer. Unlike the generator, a LeakyReLU layer was chosen as an activation layer in the discriminator network. Batch normalization and LeakyReLU layers were changed with the Sigmoid and Flatten layers in the last block of the discriminator. The parameters of both networks are given in Table 2 and Table 3. The Adam optimizer was used to train the network. The learning rate was equal to 0.0002. The decay factor for the first momentum—β_1—and the decay factor for infinity norm—β_2—were equal to 0.5 and 0.999, respectively.

### 3.3. Dataset

**Figure 3:**
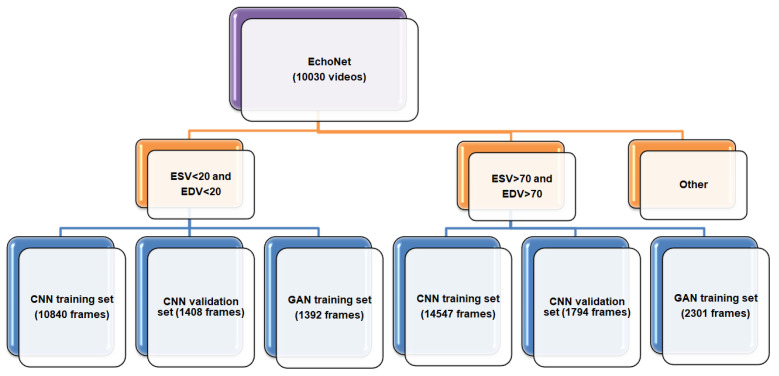
Distributed dataset.

EchoNet (dynamic cardiac ultrasound database) was used to train the GAN network and classification Convolutional neural network (CNN) [36]. This dataset consists of echocardiogram videos from 10,030 individual patients. Although the videos were not classified by disease, they provide information about the dimensions and some parameters of the presented heart. More specifically, a separate CSV file contains information about the ejection fraction (EF), end-systolic volume (ESV), and end-diastolic volume (EDV) values of the heart in each video. These echo videos contain only an apical-4-chamber (A4C) view of the heart, and their resolution size is 112 × 112. Then, we put appropriate files from the dataset into two folders (i.e., classes) according to the heart’s ESV and EDV values. It is known that the EDV of some hearts corresponds to the ESV of others. Therefore, when the videos are divided into frames, the data of two sets can be intersected. For this reason, we put the hearts’ videos where ESV < 20 and EDV < 2 into the first folder/class, and the hearts’ videos where ESV > 70 and EDV > 70 were put into the second folder/class in Figure 3. In this paper, we aimed to train two networks: GAN and CNN.

The first is a GAN network for generating artificial images. The second is the CNN network, which will be used in the proposed method. Therefore, after dividing the data into folders according to their size, the files in each folder were divided into 3 parts in the 8:1:1 ratio. Notably, 8× parts of data were taken for training the CNN network, 1× part was taken for the validation of the CNN network, and 1x part was taken for training the GAN network. After that, the videos were divided into frames and placed in appropriate folders. This distributed dataset is graphically illustrated in Figure 3.

### 3.4. Training the GAN Network and the Results

**Figure 4:**
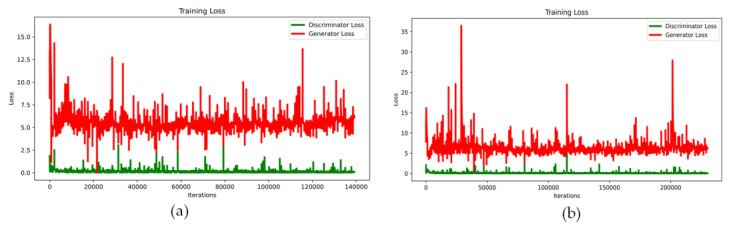
The value of losses during the training process: (**a**) Heart20 training loss and (**b**) Heart70 training loss.

**Figure 5:**
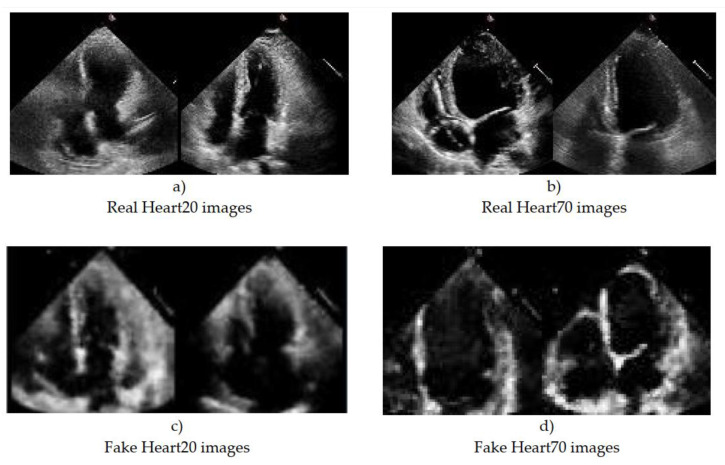
The A4C real and synthetic echo images of the heart generated by the GAN architecture.

Training a GAN network is a very complex process, and it is trained over very long iterations. Our goal was to train images Belonging to two different classes: Class 1 is a class of heart images with ESV < 20 and EDV < 20, which is conditionally called Heart20. Class 2 is the class of cardiac images with ESV > 70 and EDV > 70, which is conditionally called Heart70. In this case, the GAN network should be trained twice. The generated images were controlled/judged at each epoch, and training was stopped if the images’ quality was deemed satisfactory. Although the Heart20 data are less than the Heart70 data, the GAN network was able to draw images of the class Heart20 faster and with better quality. Therefore, during the generating of Heart20, training was stopped earlier in Figure 4. The real images in the Heart20 and Heart70 datasets and appropriate the GAN-generated synthetic images Figure 5.

## 4. Proposed Evaluation Method

We have proposed a two-step evaluation approach and an appropriate new evaluation metric method for the images generated by the GAN. The first step is to evaluate the quality of images during the training. For this, a new measure similar to the FID score, but faster and easier to calculate, should be used. This method mainly evaluates the quality of the images and the similarity of the synthetic images with the real images used for training. This will be discussed in detail in Section 4.1. The second step is to evaluate the images generated by the trained GAN. We have proposed a new method—using the CNN network—for this step. This method evaluates the diversity of the generated datasets and their proximity to other real images. This will be discussed in detail in Section 5.

### 4.1. Problem Statement

Evaluating the network performance and comparing the results using the GAN architecture is a highly complex task. Usually, reference-based and non-reference-based methods are used to estimate the distance between different distributions and to evaluate the performance of the network. The most common methods are the assessment of IS and FID scores.

As mentioned above, IS method uses a pre-trained InceptionV3 model to evaluate the quality and diversity of the generated synthetic images. The InceptionV3 model was trained using the ImageNet database, which contains more than one million images. The InceptionV3 model can classify images into 1000 classes with an accuracy of 78.8%. However, the IS cannot evaluate the similarity of the generated images to the real ones. Therefore, a new FID method was proposed to evaluate the quality of images. The FID method compares the distribution of the generated synthetic images with that of the real images used to train the generator. For this, the feature map of the last average pooling layer of the InceptionV3 network is used. This layer consists of 2048 neurons. Based on the sets of feature maps generated in the last layer when the synthetic and real images are fed into the network, the FID score is determined using Equation (3) as follows:

(3) $$d^{2}\left(F,G\right)={\left|\mu_{x}-\mu_{y}\right|}^{2}+tr[\Sigma_{x}-\Sigma_{y}-{2(\Sigma_{x}\Sigma_{y})}^{1/2}]$$

Here, μ_x_ and μ_y_ are the average values of the activations A(x_i_) and A(y_i_) generated in the last average pooling layer when real and synthetic images—(x_i_, y_i_) are fed to the InceptionV3 network. Then, $\Sigma_{x}\Sigma_{y}$ are the sample covariance matrices of these activations. The trace of this matrix is determined using Equation (4):

(4) $$tr\left(\sqrt{\Sigma_{1}\Sigma_{2}}\right)=\sum_{i=1}^{m-1}\left|\sqrt{\lambda_{i}(C_{1}^{T}C_{2}C_{2}^{T}C_{1})}\right|$$

In this case, the time complexity for calculating the eigenvalue of $\left(C_{1}^{T}C_{2}C_{2}^{T}C_{1}\right)$ was determined as $O(mdn+m^{2}n+m^{3})$, and if the number of samples is large, the time complexity is $\left(d^{2}m+m^{3}\right)$. Here, m and n are the numbers of real and synthetic samples, respectively, and d is the number of neurons in the last layer. If m<<d, the time spent to produce a small number of synthetic images is proportional to the number of neurons, and if d<<m with a large amount of data, it is a quadratic function. In [33], the authors presented a new approach to overcome this issue, and the time complexity of their proposed method was $\left(d^{3}+d^{2}m\right)$. Therefore, when working with a large amount of data, the second method is more convenient than the first, where the time required depends more on d than on the number of samples. However, considering that this relationship is cubic, by reducing the value of d, theoretically, we can significantly reduce the time required to calculate the FID value. In particular, in the case when the FID value is used as an additional loss value, this method plays an important role in network training, as it drastically increases the training speed. Although the computation time of the fast FID method is much shorter than the original FID method, as noted in the same paper, it would be desirable to further reduce this time because, usually, GAN networks are trained for a long time, and in cases where the FID value is used as an additional loss, the total training time can be significantly reduced by fast calculating the FID value at each epoch. The memory required for training would also be reduced. For this reason, we have proposed a method with the possibility of fast calculation. 

In addition, the InceptionV3 network is only designed to work with images of people and objects and not with medical images and videos. Medical images, especially echocardiograms, have special aspects which are different from ordinary images:

- They are noisier and blurrier than ordinary images;
- The edges are not clearly defined;
- Usually generated synthetic echo images are of gray-scale quality, i.e., mostly single-channel.

In this situation, instead of the InceptionV3 network, the FID estimation accuracy can be improved by using a network that can better classify medical data. For example, in [38], a special method was designed for video quality estimation. However, no specific method has been proposed to assess echocardiogram images. 

### 4.2. Method Description

The main reason for the time-consuming calculation of the FID score is that the InceptionV3 network has many parameters to calculate because, after feeding one image to the network, from the input to the last average pooling layer, a total of 21,785,568 parameters are required for the calculation. In addition, calculating the Fréchet distance, which is used to calculate the FID score, also takes a lot of time. As a *2048xbatch_size* matrix is generated from the Adaptive average pooling layer. Therefore, in the process of FID calculations, instead of the InceptionV3 network, we proposed to use a network with fewer parameters and designed for the classification of echo images, given in Table 4. Furthermore, the size of the last layer which will be used in the calculation of Fréchet distance also will be smaller than that of the InceptionV3 network, i.e., than the last Adaptive Average Pooling layer.

For this purpose, we built a CNN architecture that can classify echocardiogram images with high accuracy. The parameters of the architecture are listed in Table 4. The output of the last convolution layer is used to calculate the Fréchet distance. More precisely, the parameters from the ***Input layer to the last ReLU layer are used***. Then, a total of 25,782 calculations are required to obtain the activation—A(x_i_) of a single image. This means 844.99 times fewer parameters compared to the InceptionV3 network. In addition, a 256xbatch_size matrix is generated from the last convolution layer. Its size is also 4 times less than that of the last used layer (2048xbatch_size) of Inception. 

As mentioned earlier, the EchoNet dataset was used to train this network. To avoid errors in the evaluation of the network, the dataset was divided into a certain ratio. One part was reserved for training GANs and the rest was used for training and validating CNN. That is, the CNN network cannot see the set of images intended for generating synthetic images. In addition, a dropout layer was added after each convolutional block to prevent network overfitting.

The CNN was trained for 30 epochs. The Adam optimizer was used as the optimization algorithm and the decay factor for the first momentum—$\beta_{1}$—and the decay factor for the infinity norm—$\beta_{2}$—was set to 0.95 and 0.99, respectively. This is because, in our previous work, it was found that these values affect high performance [2]. The validation accuracy of the network reached 90.75%. The advantage of this network over InceptionV3 is that it is adapted to gray-scale images, rather than to RGB images. This ensures that the quality of the images was maintained during the assessment process. Another advantage of this network is that it is trained to extract special features of echo images. This network is called MedicalNet, and the method that assesses the quality of the synthetic images is called the FMD method. 

### 4.3. Experimental Results

After that, we generated images of Heart20 using the trained GAN network and determined their FID and FMD values. To determine the reliability of the estimation methods, we checked them on two different databases: the dataset used to train the GAN and the validation dataset used to evaluate the CNN network. In order to estimate the distribution across the group, we set the batch size equal to the size of these datasets, i.e., 1392 and 1408, respectively. Since the synthetic images were generated based on the GAN training dataset, the FID and FMD values were expected to be smaller. Because synthetic images will be more similar to this set. Even the CNN validation dataset is unfamiliar for synthetic images; they should have small enough FID and FMD values, as they also belong to the same class as the GAN training set. That is, synthetic images should be similar to every real image of the same group, despite their usage in the training process. As can be seen from Table 5, the real synthetic images are more similar to the GAN training set. The important point here is that the value of FMD is slightly higher than the value of FID. However, when evaluating the similarity to the validation set, the value of FMD is much higher than the value of FID. 

As can be seen from Table 5, the speed of calculating the FMD value is significantly higher than that of the FID value. To further validate this, we performed the following experiment. The calculation time of both methods was measured for different batch sizes. The times required to calculate the FID and FMD values obtained in the experiment are listed in Table 6.

As shown in Table 6, our proposed method required up to 107.9 times less computation time than the conventional FID methods. Several fast algorithms have been proposed to reduce the time required to compute the FID value [48]. Comparative results of the proposed method and FID method were given in Table 6. 

The speed of our proposed algorithm was higher than that of the other algorithms. Authors [49] found that the evaluation time of eight images using the fastFID method was up to 13 times faster than using the normal FID method. Our approach required 86.63 times less time than the FID method. The fastFID algorithm took 2.8 times less time to evaluate 128 images than the FID method, whereas our algorithm required 99.6 times less time than the FID method for this process. That means our proposed algorithm is much more (up to 35.5 times) faster than the fastFID method. 

Now, it may be assumed that this difference affects training time if the FID, fast FID, and FMD scores are used in the GAN network as an additional loss. The loss function will be in the following form (5):

(5) $$G*,D*=\arg{min}_{G}{man}_{G}L_{CGAN}(G,D)+\lambda L_{L1}(G)$$

During the training, the FID loss is calculated at each iteration. If the number of images in the dataset is *n* and the batch size is *b*, the number of iterations in each period is expressed as *n/b,* and for *m* epochs, it will be determined as N*_i_* = m∗n/b. Then, the additional time required to calculate the FID value will be expressed as follows:

(6) $$t=N_{t}*t_{0}$$

**Figure 6:**
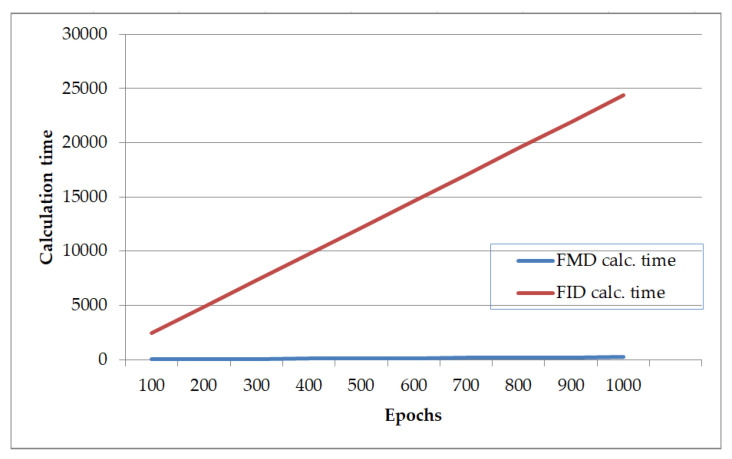
Time required for calculating the additional loss value in 1000 epochs with 128 batch.

Here, t_0_ is the time spent calculating the FID value in a single batch. Using the above formula, in Table 5 and Figure 6, we show a comparison of excess calculation time for different epochs when the batch size is equal to 128. We may take iterations per epoch equal to 10.

Figure 6 shows that using the FMD value as an additional loss for training the GAN (with 128 batch size dataset) during 1000 epoch requires 24,127 fewer seconds than using the FID value; that is, approximately 6.7 h can be saved. Moreover, it can be seen that the computation time of the FID value in the FastFID method is almost the same as the computation time of the FMD value. However, in the proposed approach, we used SCIPY.LINALG.SQRT function for calculating the FMD values. If the matrix trace is calculated instead of using the SCIPY.LINALG.SQRT function as mentioned in [49,50], the time required to calculate the FMD value can be reduced further by a factor of 100. 

### 4.4. New CNN-Based Evaluation Method

This method can only be used after the GAN network is fully or sufficiently trained, as this method requires a large number of synthetic images. This method works as follows.

After the GAN network is trained, a large dataset of synthetic images will be generated using the trained GAN. The CNN network is trained using this synthetic dataset. Real images are used as a validation dataset. These real images can be images used in the training GAN or other images of the same classes. However, the most important thing is to pay attention to the fact that the size of the training and validation dataset should be in a 9:1 or 8:1 ratio. So it is recommended to develop a dataset of synthetic images based on the number of available real images. 

Another important thing to mention is that, as CNN is being used here for classification purposes, we need images of at least two classes. This means that we need to train the GAN network twice, that is, we need to train it separately to generate images belonging to two different classes.

**Figure 7:**
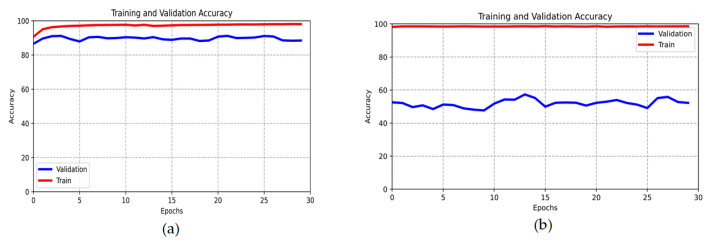
Training and validation accuracy of real (**a**) and synthetic (**b**) datasets.

Because the following conclusions can be drawn in Figure 7, which serves as our main evaluation tool. In our case, images belonging to two different classes (Heart20 and Heart70) were generated using the GAN network. We had 2301 and 1392 images in each class, respectively, for training the GAN network. So we generated 18.000 Heart70 synthetic images and 11.000 Heart20 synthetic images. For this task, we used the network used in the FMD metric (see Section 4.2.) Then we trained the CNN network. The training and validation accuracy of the network for synthetic and real training datasets is shown in Figure 7.

The rapid rise of the training line to 100% means that the homogeneity of the synthetic images is very high. In particular, the fact that the training line reaches 100% in the initial epochs, and the validation line takes very low (30–40) values means that almost all synthetic images are the same, that is, mode collapse or overfitting in the GAN network.

Depending on the maximum value of the validation line, synthetic images can be evaluated as the 5-point Likert scale, as follows:

- Less than 40—Very bad. Low quality or the same images, mode collapse, or overfitting occurs in GAN.
- 30–50—Bad. The diversity is very low. Most images are unrealistic and of poor quality.
- 50–70—Satisfactory. The diversity is low. Some images are unrealistic and of low quality.
- 70–80—Good-quality images. The diversity is high, but there are still some disturbances in some images.
- More than 80—Much like the real images, the diversity is very high.

We can see from Figure 7 that the generated images are satisfactory but of poor quality. In fact, there were also very noisy images within the dataset. In addition, it could be seen that many images are very similar to each other. That is why the training line was close to 100% from the earlier epochs.

To validate the algorithm’s effectiveness, this study conducted numerous tests using accuracy, recall, F-measure (FM), and AP evaluation metrics are the key indices for gauging the accuracy of GAN models. Samples in the binary classification issue can be classified as true positives (TP), false positives (FP), true negatives (TN), or false negatives (FN), depending on the relationship between the actual and expected categories (FN) The confusion matrix of the categorization is displayed in Table 7.

The F-measure (*FM*), which balances the precision and recall rates and measures the weighted average, was tested. This rating considers both the true positive and false negative rates. The *FM* is the characteristic that detects an object most frequently because it is challenging to measure the accuracy rate. False negatives and true positives performed better in a detection model that used the same weight. Precision and recall, however, must be considered if real positives and false negatives are different. The ratio of genuine positive observations is known as precision. The average precision and recall rates of our suggested method can be calculated as illustrated in Equations (7) and (8):

(7) $$Precision=\frac{TP}{TP+FP}$$

(8) $$Recall=\frac{TP}{TP+FN}$$

When the accuracy ratio is plotted against the recall rate, the resulting graph is called a precision–recall curve (P-R plot). The effectiveness of the model may also be determined by its *FM* score.

The score can be defined as follows:

(9) $$FM=\frac{2\times precision\times recall}{precision+recall}$$

The *FM* average accuracy of each detection was also employed as a criterion in this investigation (AP). The following is a definition of the term:

AP = Precision (Recall)*d*(Recall) 1

Table 8 shows the comparison of the three models’ performance of the generated synthetic medical image efficiencies. Our proposed approach model outperforms competing models in terms of both accuracy and recall rate, with an accuracy of, at most, 98.71 percent.

## 5. Discussion

Medical images are used to diagnose diseases, which means that their quality can affect human life and health. So the correct evaluation of the quality of synthetic medical images is more important than the evaluation of ordinary images. For this reason, in recent years, many methods and metrics for assessing the quality of synthetic medical images have been proposed. However, most of them are mainly methods adapted for the evaluation of MRI, CT, and PET images. According to the information we have, no special method for evaluating echo images has been developed. Generally, MRI, PET, and CT images are very different from echo images. MRI images are 3D images and have less noise than echo images. Therefore, the MAE, MSE, and SSIM methods that are used effectively for MRI and PET images will be ineffective for echo images. For this reason, the most recent published studies on echo imaging have mainly used the FID value. However, the FID value only compares the distribution of the synthetic images with the distribution of the training set of images. However, if the twins of the real images are generated due to the mode collapse or overfitting of the GAN, the FID value will be still very small; i.e., these images are estimated as good-quality images. It can be seen that the FID value cannot assess whether the network is overfitting. Moreover, although generated images are in high resolution and are not identical to real images, they may reflect another disease. This is also a big problem, as generated images are usually used in classification problems. However, the FID metric cannot estimate this phenomenon.

The proposed CNN evaluation method, unlike it, can evaluate the variety of images, not only how close they are to real images but also how different they are from another class. It can also assess whether the GAN network is working incorrectly, reproducing the same images over and over again, low image quality, and how close the distribution of synthetic images is to other real images that were not used to train the GAN.

The intervention of medical personnel was not used to evaluate the accuracy of this work. However, non-overlapping datasets were used to evaluate the proposed method. Specifically, the real images used to train the GAN were not used to evaluate the CNN trained on synthetic images. That is, the similarity of synthetic images not only with the trained set but also with the set outside of it was evaluated.

Of course, the proposed CNN method is time-consuming, but, once it is trained, it can evaluate as many images as desired in a very short time. In addition, it can estimate synthetic images from two or more classes at once. However, in this process, it can estimate how different the distributions of synthetic images of different classes are. Yet the FID metric does not have such capabilities. In addition, last but not least, synthetic images are usually used for classification purposes. Therefore, evaluating them using the CNN method also evaluates how satisfactory these images are for classification problems.

Since the CNN method is a method that can only be used after the GAN training is completed, by using the FMD method during the GAN training, the quality of the images can be evaluated during the training as well. Since the FMD method is much faster than the FID method, monitoring the quality of the images by evaluating the FMD at each epoch does not bother much the training process.

Using the CNN method together with the FMD method allows us to quickly and reliably evaluate the images generated by the GAN network from the initial training stage to the stage after training.

## 6. Future Direction

Loss is a value that represents the summation of errors in existing models. Errors occurred mainly because of two major problems, namely, mode collapse and non-convergence. One feasible method to make GAN solve these two challenges is to redesign the network architecture to obtain a more powerful model. Accuracy measures how well our model predicts by comparing the model predictions with the true values in terms of percentage. Having a low accuracy but a high loss would mean that the model makes great errors in most of the data. However, if both loss and accuracy are low, it means the model makes small errors in most of the data. However, if they are both high, it makes big errors in some of the data. Finally, if the accuracy is high and the loss is low, then the model makes small errors for just some of the data, which would be the ideal case [53,54,55]. The evaluation of images using the CNN network is qualitative rather than quantitative, so further research should be conducted to evaluate images quantitatively based on the accuracy graph of the training and validation of the CNN network [56,57]. 

## 7. Conclusions

In this study, we developed a specific FMD metric and CNN method for the evaluation of synthetic echo images. It is recommended to use a combination of these methods to evaluate the generated images during and after the training process of the generative network. This FMD metric is easier to calculate than the FID method. Especially when using it as an additional Loss function in GAN networks, the difference will be great.

The evaluation of synthetic images using the CNN network has the following advantages that are not available in other evaluation methods:

- It can evaluate the quality of synthetic images belonging to two or more classes at the same time.
- It is possible to evaluate the diversity of the generated Images and the presence of the same images.
- It can estimate how close the distribution distance of synthetic images is to that of real images of the same class and how far it is from that of other classes.

## Figures and Tables

**Table 2:** The parameters of the Generator architecture.

No.	Names of the Layers	Number of Convolutional Layer Filters	Convolutional Layer Filter Size/Stride/Padding
1.	Input + Reshape		
2.	ConvTranspose2d + BatchNorm + ReLU	512	4/1/0
3.	ConvTranspose2d + BatchNorm + ReLU	256	4/2/1
4.	ConvTranspose2d + BatchNorm + ReLU	128	4/2/1
5.	ConvTranspose2d + BatchNorm + ReLU	64	4/2/1
6.	ConvTranspose2d + Tanh	1	4/2/1

**Table 3:** The parameters of the Discriminator architecture.

No.	Names of the Layers	Number of Convolutional Layer Filters	Convolutional Layer Filter Size/Stride/Padding
7.	Input		
8.	Conv2d + BatchNorm + LeakyReLU(0.2)	64	4/2/1
9.	Conv2d + BatchNorm + LeakyReLU(0.2)	128	4/2/1
10.	Conv2d + BatchNorm + LeakyReLU(0.2)	256	4/2/1
11.	Conv2d + BatchNorm + LeakyReLU(0.2)	512	4/2/1
12.	Conv2d + Sigmoid + Flatten	1	4/1/0

**Table 4:** The parameters of the GAN architecture.

No.	Names of the Layers	Number of Convolutional Layer Filters	Convolutional Layer Filter Size/Stride/Padding	Dropout (%)
1.	Input			
2.	Conv2d + BatchNorm + ReLU + Dropout	256	4/2/1	20
3.	Conv2d + BatchNorm + ReLU + Dropout	2	4/2/1	20
4.	Conv2d + BatchNorm + ReLU + Dropout	128	4/2/1	20
5.	Conv2d + BatchNorm + ReLU + Dropout	16	2/1/0	20
6.	Output			

**Table 5:** FID and FMD values for different datasets and time required to calculate them.

Batch Size	FID		FMD		Real Datasets Name
	Time	Value	Time	Value	
Heart20 fake dataset					
1392	253.372	34.41	1.813	16.62	GAN training set
1408	313.716	42.56	2.456	29.45	CNN validation set
Heart70 fake dataset					
1794	308.225	62.81	1.930	138.38	GAN training set
2301	326.37	61.04	2.120	129.23	CNN validation Set

**Table 6:** Time required for calculating the FID and FMD values.

Batch	FMD Time	FID Time	FID/FMD Ratio
8	17.43 ms	1.51 s	86,632.24
16	37.559 ms	3.468 s	92,334.73
32	62.775 ms	6.226 s	99,179.61
64	112.565 ms	12.143 s	107,875.4
128	244.611 ms	24.371 s	99,631.66

**Table 7:** The confusion matrix of the real and predicted categories.

Labeled Name	Predicted	Confusion Matrix
Positive	Positive	TP
Positive	Negative	FN
Negative	Positive	FP
Negative	Negative	TP

**Table 8:** Comparison of the proposed model with other state-of-the-art GAN methods for generating synthetic medical images.

	Deep Pix2Pix GAN [51]	MRI via GAN [52]	Proposed Approach
Precisions	96.35%	93.92%	98.71%
Recalls	64.98%	75.28%	82.13%
mAP	41.12%	38.03%	29.49%

## Data Availability

Not applicable.
